# Mechanical Performance and Corrosion Behaviour of Diffusion-Bonded A5083 Aluminium and A36 Mild Steel with Gallium Interlayer

**DOI:** 10.3390/ma15186331

**Published:** 2022-09-13

**Authors:** Asmawi Ismail, Nurul Husna Othman, Mazli Mustapha, Mohamed Shuaib Mohamed Saheed, Zaki Abdullah, Musa Muhammed, Asmalina Mohamed Saat, Faizal Mustapha

**Affiliations:** 1 Department of Mechanical Engineering, Universiti Teknologi PETRONAS, Seri Iskandar 32610, Malaysia; 2 Department of Maritime Engineering Technology (MET), Malaysian Institute of Marine Engineering Technology, Universiti Kuala Lumpur, Lumut 32200, Malaysia; 3 Department of Materials Engineering, University of Quebec, 555 Bd de l’Université, Chicoutimi, QC G7H 2B1, Canada; 4 Department of Marine and Electrical Engineering Technology (MEET), Malaysian Institute of Marine Engineering Technology, Universiti Kuala Lumpur, Lumut 32200, Malaysia; 5 Department of Aerospace Engineering, Faculty of Engineering, Universiti Putra Malaysia, Serdang 43400, Malaysia

**Keywords:** diffusion bonding, dissimilar joining, aluminium alloy, mild steel, gallium, corrosion rate

## Abstract

This article investigated the mechanical performance and corrosion behaviour of a diffusion-bonded A5083 aluminium/A36 mild steel dissimilar joint with a Gallium (Ga) interlayer. The bonding parameters were the bonding temperature (525 and 550 °C), holding time (60 and 120 min) and surface roughness (800 and 1200 grit). Property characterisation was achieved using Scanning Electron Microscopy (SEM), Energy Dispersive X-ray (EDX) analysis, Vickers microhardness tester, Izod impact tester and potentiodynamic polarisation testing. The results revealed that the significance of the bonding parameters was in the order bonding temperature > surface roughness > holding time. Increasing the bonding temperature resulted in an increase in the impact strength and a corresponding reduction in the corrosion rate and microhardness. However, increasing the grit size decreased the microhardness and a corresponding increase in the impact strength and corrosion rate. The impact strength and corrosion rate decreased with the increasing holding time while the microhardness followed a reverse trend. It was also discovered that incorporating the Ga interlayer resulted in a 67.9% improvement in the degradation rate.

## 1. Introduction

The technology of dissimilar metal joining has a variety of applications, especially when a certain property combination is desired. These joints have received more attention from researchers in recent decades as they create the possibility of achieving a compromise between cost and improved joint efficiency [1]. However, creating such joints poses more challenges due to the differences in the properties of the participating Base Metals (BMs) [2,3,4]. Diffusion bonding involves bringing the participating BMs within interatomic distances by applying pressure at elevated temperatures. The quality of joints, therefore, depends on the pressure, temperature and holding time, among others. For instance, the ultimate tensile strength of titanium alloy/austenitic stainless steel joint fluctuated as the temperature, heating rate and bonding pressure gradually increased [3,4]. Moreover, it was discovered that increasing the bonding temperature improved the corrosion resistance of the stainless steel joints [5]. Additionally, the surface roughness of the BMs prior to bonding has also been reported to considerably affect the quality of the diffusion-bonded joints [6]. Moreover, an extensive review of the effect of bonding parameters on the properties of dissimilar joints involving stainless steel and other metals was recently documented by AlHazaa and Haneklaus [7].

Diffusion bonding applications include the marine industry and nuclear power plants, among others [5,6,7,8,9,10]. Before now, structural components for marine applications have been fabricated using mostly fusion joining techniques, while some studies have documented the use of composites [11]. However, compared to other fusion joining techniques, diffusion-bonded dissimilar joints are less prone to interfacial failure due to the reduction in Intermetallic Compounds (IMCs) formation [11,12,13,14]. Among the efforts made by experimenters in improving the quality of dissimilar joints is the incorporation of an interlayer between the BMs. Several interlayers including aluminium, copper, zinc, nickel, titanium, molybdenum and Carbon Fibre-Reinforced Polymer (CFRP) have been reported [15,16,17]. A suitable interlayer has the ability of retarding oxide formation and facilitates the release of residual stresses between the BMs [18].

Despite the existence of several dissimilar metal joints, aluminium/mild steel joints have received more attention from researchers, particularly in the marine industry. This is due to the desire to reduce the overall weight of the deckhouse superstructure, energy consumption and cost of production [18,19]. These joints combine the strength and wear resistance conferred by the low carbon steel alongside the corrosion resistance and lightweight offered by the aluminium alloy, which adapts the joint to the adverse marine environment [2,20,21]. Ga, conversely, is a very ductile metal with a melting point relatively lower than that of mild steel and aluminium [12]. Moreover, Shirzadi et al. were one of the pioneer researchers to adopt a Ga interlayer to produce diffusion-bonded joints [22,23]. In a more recent study, Shirzadi et al. also reported that adopting a Ga interlayer improved the tensile strength in AISI 304L and ASTM B265 titanium dissimilar joints [24].

Over time, the advancement has been made in creating dissimilar joints involving aluminium and/or steel with or without interlayers using diffusion bonding. Abdul Rahim et al. [25] reported that joints produced in a vacuum exhibit superior tensile properties relative to those produced under atmospheric conditions due to the reduction in the contamination of the faying surfaces by atmospheric oxidation. Later on, Pan et al. [13] incorporated multiple interlayers to produce 304L stainless steel/Zircaloy-4 joints and observed that the bonding parameters did not significantly influence the interfacial microhardness despite IMC formation. In another study, Qin et al. [26] recommended intermediate bonding conditions if the optimum joint quality is desired after conducting a study on titanium/stainless steel dissimilar joints. In a more recent study, Choy et al. [27] reported that increasing the IMC layer thickness has a detrimental effect on the joint’s strength.

Furthermore, catastrophic failures owing to corrosion have also been reported in the marine industry. The damage is more severe at high galvanic potentials between the participating BMs [22,28,29,30]. However, the degradation rate is also influenced by the nature of the environment, as a corrosive environment such as seawater further accelerates the process [23,24]. In addition, other factors such as exposure time, the concentration of inhibitors and microstructural modification also significantly affect the corrosion integrity of the joints [25,27,31,32]. Lately, the use of the potentiodynamic polarisation technique to measure the rate of degradation has received attention from experimenters as it creates the possibility of the instantaneous determination of the corrosion rate and eases the detection of localised corrosion [33].

From the foregoing, it is evident that the quality of diffusion-bonded joints is greatly dependent on the bonding parameter. Furthermore, though several studies on aluminium/mild steel dissimilar joints exist, little or no study has been conducted to investigate the properties of the dissimilar joint with a Ga interlayer despite having the potential to improve the overall joint quality. The dissimilar joints also form part of the marine industry’s structural component (deck house superstructure). This study explored the feasibility of creating aluminium/mild steel dissimilar joints with a Ga interlayer. The effect of the bonding temperature, surface roughness and holding time on the joint’s mechanical properties and corrosion behaviour were also investigated. The effect of these parameters on the joint morphology and the relationship between the elemental composition of the reaction layer and weld quality parameters were also ascertained.

## 2. Materials and Methods

### 2.1. Materials

The BMs used for this study are bar-shaped A36 mild steel and AA5083 aluminium alloy with dimensions of 10 mm × 10 mm square bars. The chemical compositions of the BMs are presented in Table 1.

### 2.2. Diffusion Bonding of the BMs

**Figure 1:**
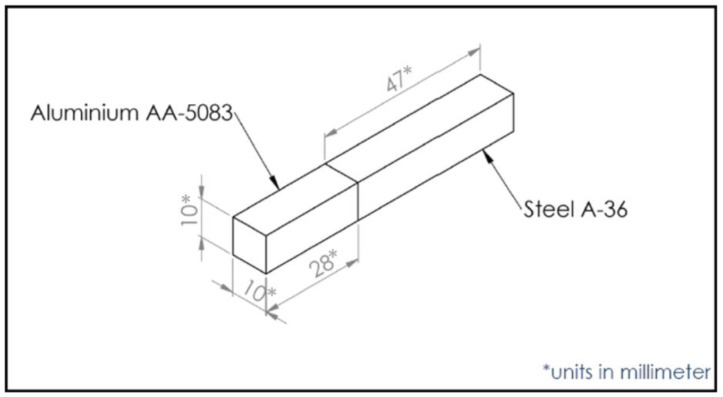
Dimension and joint configuration of the diffusion-bonded mild steel and aluminium.

**Figure 2:**
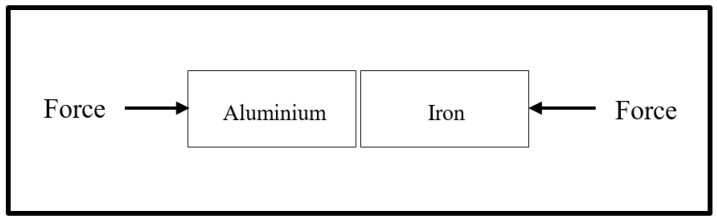
Diagram showing the direction of pressure application.

Figure 1 shows the dimension and joint configuration of the BMs. The bar-shaped BMs to be joined were first polished to eliminate oxides at the faying surfaces. Polishing was achieved using an emery paper with grit sizes of 800 and 1200. Afterwards, the polished surfaces were cleaned with acetone and dried with hot air. Next, a thin film of Gallium was manually applied to the faying surfaces and subsequently held in intimate contact with a clamp assembly at a pressure of 7 MPa, as illustrated in Figure 2. The bonding pressure was achieved by applying a pre-pressed pressure to the clamp assembly using a hydraulic press machine.

In order to highlight the influence of the Ga interlayer on the joint properties, some samples were fused directly without Ga coating. The clamped assembly was then heated in a programmable electric furnace varying the heating temperature between 525 °C and 550 °C at a constant heating rate of 5 °C/min. The holding time was also varied between 60 min and 120 min. After the heating process, the samples were slowly cooled in the furnace. To prevent the BMs from sticking to the clamp, the surfaces between the BMs and the clamp were smeared with boron nitride [8]. Table 2 shows the detailed conditions of the different samples taken into consideration.

### 2.3. Microstructural and Mechanical Characterisation

Mechanical characterisation of the joints was achieved by conducting the Izod Impact test and Vickers microhardness test. The microhardness testing was conducted by applying a load of 50 gf for 10 s across the bonded BMs using a Vickers microhardness tester [34]. The Impact test (Unnotched) was conducted using a Impact tester machine from Ceast Instron, Norwood, MA, USA in accordance with ASTM D4812 ISO 180 standards, with the sample supported as a cantilever beam [35]. The samples were clamped on the steel side at the bottom whilst the aluminium was left free on the top. The test was repeated three times and the impact toughness was taken as the resulting average. Morphological characterisation, on the other hand, was achieved using SEM and EDX analysis. A cross sectional cut was made through the joint using a waterjet Computer Numerically Controlled (CNC) cutting machine. This was followed by hot mounting using epoxy resin. Thereafter, the samples were ground using silicon carbide paper by gradually increasing the grit size from 500–2000. Subsequently, the ground samples were polished using alumina with a particle size of 0.3–0.05 μm to obtain a mirror-like surface.

### 2.4. Potentiodynamic Polarisation Test

**Figure 3:**
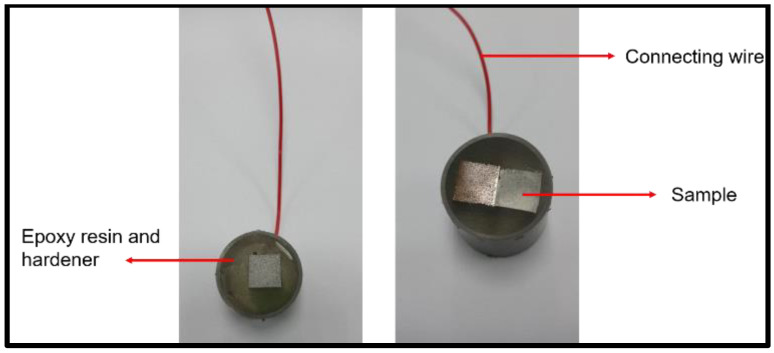
Mounted samples before the polishing process.

Potentiodynamic polarisation involves altering the potential of the metals, causing one metal to become more noble than the other through the passage of an electric current [36]. It provides crucial information regarding the sensitivity and passivity range of metals with regard to pitting corrosion [37,38,39]. It also generates a polarisation curve whose slope (Tafel slope) indicates the corrosion potential and corrosion rate at any given condition [33]. The samples for the corrosion test were sectioned into 1 cm × 1 cm (for single BMs) and 1 cm × 2 cm (for the fused samples), as depicted in Figure 3. Subsequently, an electric wire was connected to the samples by soldering followed by metallurgical preparation as before.

**Figure 4:**
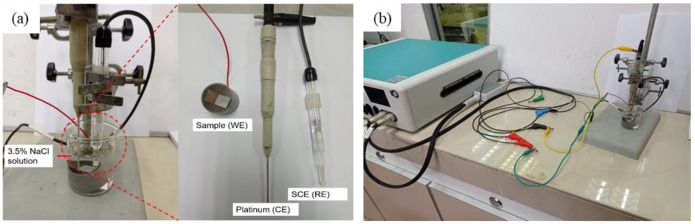
(**a**) Three-electrode electrochemical cell arrangement in the experiment with the pictures of the working electrode, counter electrode and reference electrode. (**b**) The full setup connection of the potentiodynamic polarisation testing.

The electrolyte for the immersion of the metals was 3.5% NaCl prepared by dissolving 3.5 g of NaCl powder in 100 mL of distilled water and stirred for 5 min. A potentiostat (Autolab PGSTAT 302N, Metrohm, Malaysia) was used for the potentiodynamic polarisation of the samples. The samples served as the working electrode, while the Saturated Calomel Electrode (SCE) and platinum were used as the reference and counter electrodes, respectively, at a scanning rate of 10 mV/s. The test was conducted under room conditions, as shown in Figure 4. The corrosion current density (i_corr_) measurement was calculated using the Stern–Geary equation (Equation (1)), where b_a_ represents the anodic Tafel slope, b_c_ as the cathodic Tafel slope and R_p_ as the polarisation resistance:

I_corr_ = (b_a_b_c_)/2.303R_p_(b_c_+b_a_) (1)

## 3. Results

### 3.1. Effect of Bonding Parameters on the Joint Morphology

**Figure 5:**
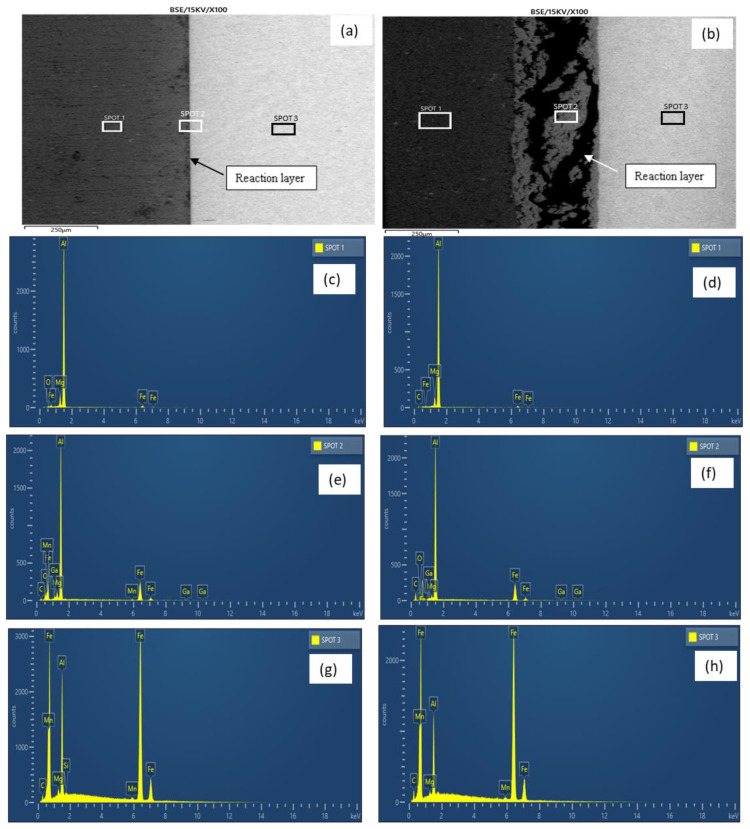
(**a**) Micrograph of sample H; (**b**) Micrograph of sample E; (**c**) Sample H EDX analysis (spot 1); (**d**) Sample E EDX analysis (spot 1); (**e**) Sample H EDX analysis (spot 2); (**f**) Sample E EDX analysis (spot 2); (**g**) sample H EDX analysis (spot 3); (**h**) sample E EDX analysis (spot 3).

Figure 5a,b show the micrographs of samples E and H, respectively. As revealed by the micrographs, increasing the bonding temperature from 525–550 °C at a constant holding time (60 min) and surface roughness (800 grit) resulted in a more distinct reaction layer with an increased thickness, as evident in Figure 5b. The elemental composition revealed by the EDX analysis (Table 3 and Figure 5c–h) also shows that a greater reduction in the BM composition (i.e., Al and Fe) was observed in sample E, with an increased bonding temperature as the increment providing the necessary energy that facilitated the diffusion of the BM elements to the reaction layer (spots 1 and 3). The diffusion of the BM atoms to the reaction layer as well as their redistribution is in accordance with Fick’s Second Law [12].

**Figure 6:**
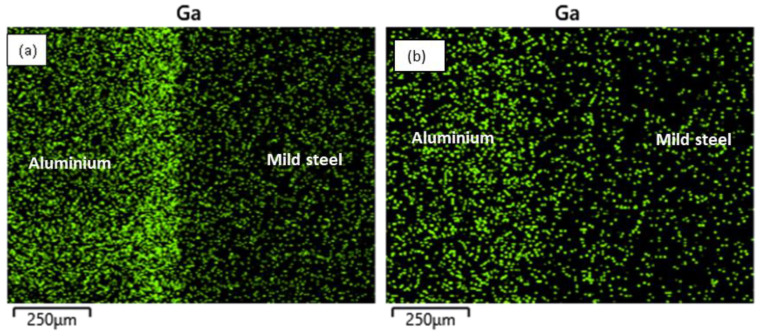
Elemental mapping of Gallium across the bonded metals: (**a**) Sample H; (**b**) Sample E.

At the reaction layer (spot 2), the composition of Ga was found to be lower with the increasing bonding temperature as the higher temperature facilitated the migration of Ga to the BM regions (Table 3). The elemental mapping of Ga across the joint is presented in Figure 6. At lower temperatures, most of the Ga appears to have migrated to the aluminium BM (Figure 6a). However, upon increasing the bonding temperature, sufficient energy was available to support the migration of the atoms to the mild steel BM region accounting for the reduction in the concentration of the Ga atoms in the aluminium BM region and a more uniform distribution of the element across the bonded BMs (Figure 6b). More so, a slight increment in the BM composition at the reaction layer was also observed with a reduction in the bonding temperature. Additionally, it was observed that the composition of aluminium (wt.%) in this region is greater than that of iron at any given time. This is due to the former’s lower potential activation energy than the latter.

**Figure 7:**
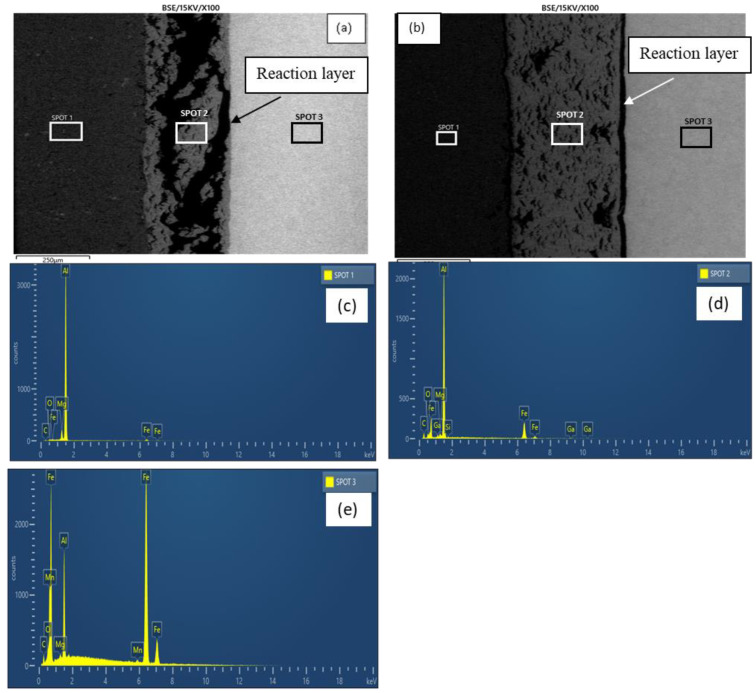
(**a**) Micrograph of sample E; (**b**) Micrograph of sample F; (**c**) Sample F EDX analysis (spot 1); (**d**) Sample F EDX analysis (spot 2); (**e**) Sample F EDX analysis (spot 3).

Figure 7a,b show the micrographs of samples E and F, respectively. As revealed by the micrographs, increasing the holding time from 60–120 min at a constant bonding temperature (550 °C) and surface roughness (800 grit) resulted in an increased reaction layer. The increment provided sufficient time that facilitated the growth of the reaction layer (Figure 7b). Additionally, a better coalescence was readily discernible at the faying surface in sample F with an increased holding time relative to sample E. This was achieved through plastic deformation accompanied by residual stress release and plastic flow between the BMs [40]. As revealed by the EDX analysis (spots 1 and 3), the holding time increment generally resulted in a slight reduction in the elemental composition of the BM regions (Table 3). Figure 7c–e show sample F’s peaks of the EDX analysis.

**Figure 8:**
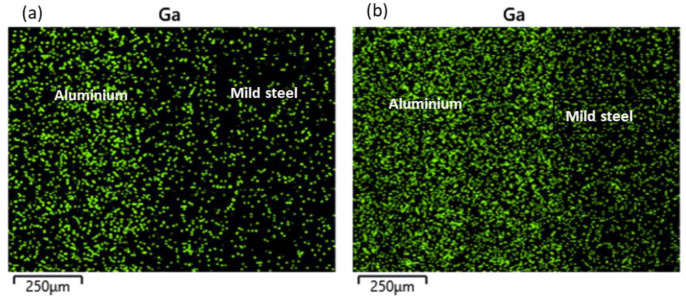
Elemental mapping of Gallium across the bonded metals: (**a**) Sample E; (**b**) Sample F.

The increment in the size of the reaction layer was accompanied by an increment in the Ga composition, as presented in Table 3 (spot 2). The elemental mapping of Ga across the joints for both samples is presented in Figure 8. From the figure, though most of the Ga appears to be concentrated in the aluminium BM region, increasing the holding time resulted in a more uniform distribution across the bonded metals (Figure 8b). Additionally, an increment was observed in the aluminium BM composition, while a slight reduction was observed in the iron BM composition of this region.

**Figure 9:**
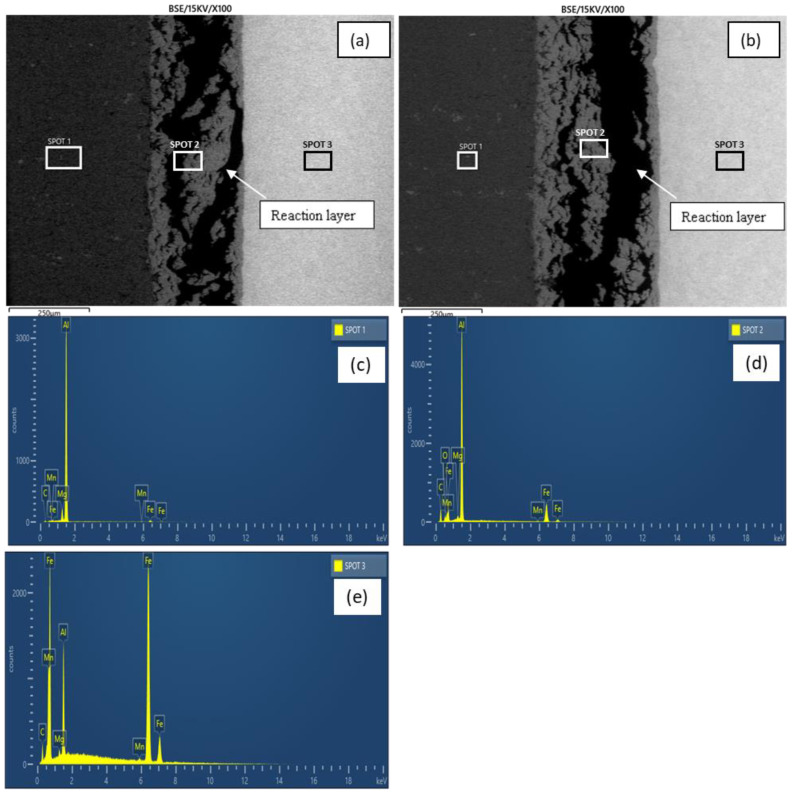
(**a**) Micrograph of sample E; (**b**) Micrograph of sample G; (**c**) Sample G EDX analysis (spot 1); (**d**) Sample G EDX analysis (spot 2); (**e**) Sample G EDX analysis (spot 3).

Figure 9a,b show the micrographs of samples E and G, respectively. As revealed by the micrographs, increasing the grit size from 800–1200 grit at a constant holding time (60 min) and bonding temperature (550 °C) resulted in a more distinct reaction layer with an increased thickness, as evident in Figure 9b. In addition, the elemental composition revealed by the EDX analysis (Table 3) also revealed a greater reduction in the BM composition in sample G with an increased grit size. Figure 9c–e show sample G’s peaks of the elemental composition.

**Figure 10:**
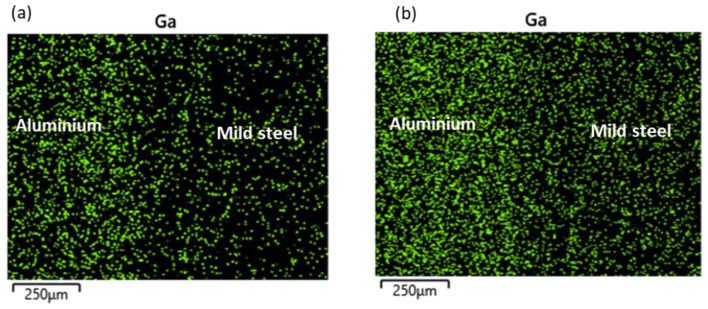
Elemental mapping of Gallium across the bonded metals: (**a**) Sample E and (**b**) Sample G.

At the reaction layer (spot 2), a reduction was observed in the BM composition and the elemental composition of Ga, as presented in Table 3. The elemental mapping of Ga across the joint is presented in Figure 10. The figure showed a more uniform distribution of the Ga across the bonded metals when the grit size was increased to 1200 (Figure 10b).

**Figure 11:**
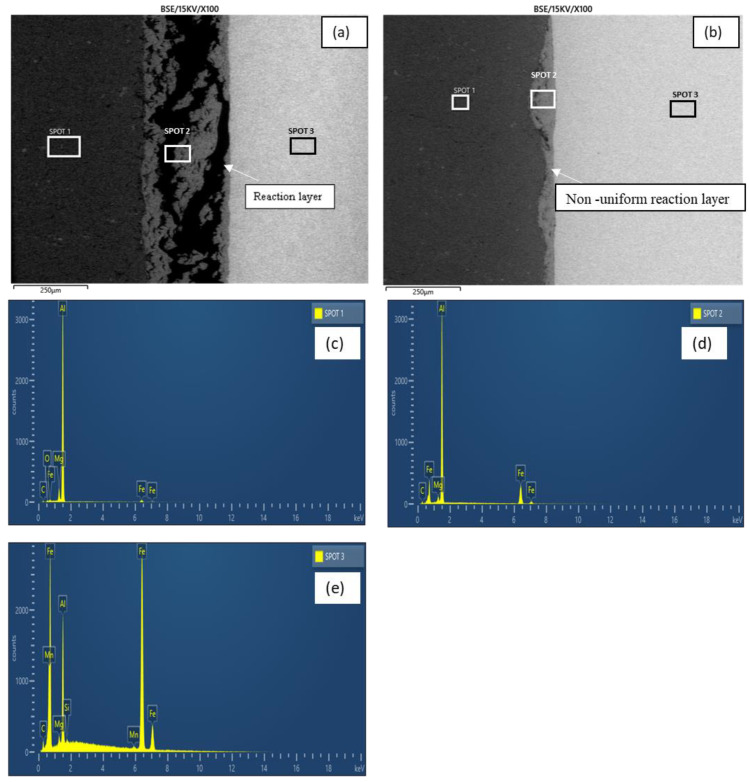
(**a**) Micrograph of sample E; (**b**) Micrograph of sample I; (**c**) Sample I EDX analysis (spot 1); (**d**) Sample I EDX analysis (spot 2); (**e**) Sample I EDX analysis (spot 3).

Figure 11a,b show the micrographs of samples E and I, respectively. An investigation of these micrographs gives a clear insight into the role of the Ga interlayer in bonding the BMs. As can be seen, the absence of Ga (Figure 11b) resulted in a thin and non-uniform bonded area. The elemental composition revealed by the EDX analysis (Table 3) also shows that a greater reduction in BM composition was observed in sample I without the Ga interlayer as its absence facilitated the diffusion of the BM atoms to the reaction layer. Figure 11c–e show the elemental composition peaks for sample I.

**Figure 12:**
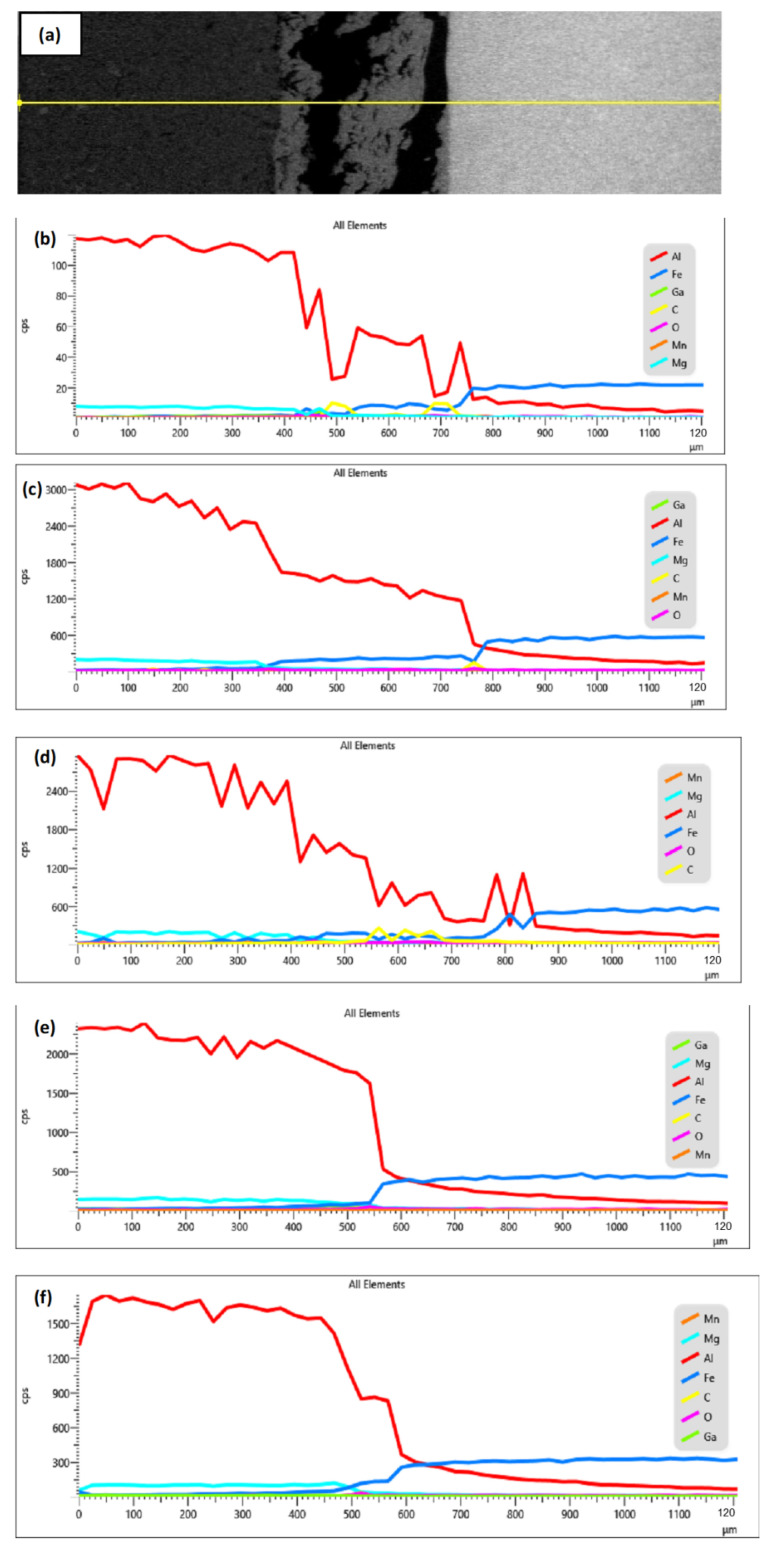
(**a**) Direction of EDX linescan; (**b**) Sample E; (**c**) Sample F; (**d**) Sample G; (**e**) Sample H; (**f**) Sample I.

Furthermore, at the reaction layer (spot 2), it was observed that the BM elemental composition in sample I without Ga was higher than in sample E, where the Ga metal was incorporated. The introduction of the interlayer metal reduced the amount of BM diffusion to the bonded area. The same occurrence was also observed regarding the diffusion of the BM atoms across the bonded area to the neighboring BM. Figure 12 shows the EDX line scan across the reaction layer for samples E–1.

### 3.2. Relationship between Joint Properties and Elemental Composition of the Reaction Layer

Table 4 presents the bonded metal’s impact strength and maximum interfacial microhardness.

**Figure 13:**
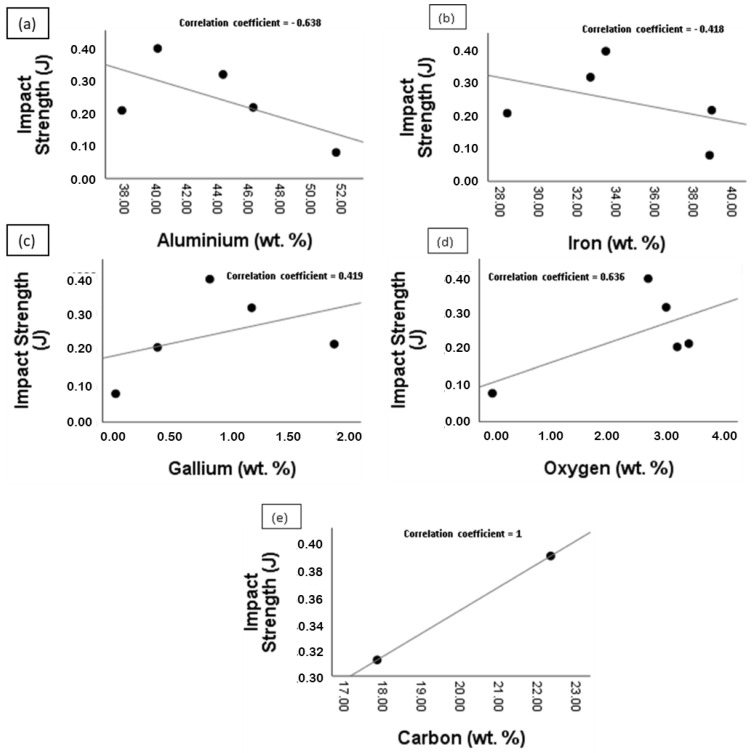
Scatter plot of the impact strength against the elemental composition (wt.%) of the reaction layer: (**a**) Impact strength against Al; (**b**) Impact strength against Fe; (**c**) Impact strength against Ga; (**d**) Impact strength against O; (**e**) Impact strength against C.

Further analysis was conducted to ascertain the relationship between the reaction layer’s elemental composition and the properties of the bonded BMs since the joint performance is dependent on the properties of this region. Figure 13 shows the scatter plot of the impact strength of the joint against the elemental composition of the reaction layer. The Al and Fe compositions (wt.%) were observed to be negatively correlated with the impact strength of the bonded metals. The correlation coefficients were found to be −0.638 and −0.418, respectively. Contrastingly, positive correlation coefficients (0.419 and 0.638) were obtained for Ga and Oxygen (O), respectively, while a perfect positive correlation (correlation coefficient of 1) was obtained for Carbon (C). The negative correlation coefficient obtained for Al and Fe is an indication that an increase in the content of these elements in the reaction layer has a tendency to reduce the impact strength of the joint. More so, the increment increases the tendency of IMC formation, which has a detrimental effect on the overall joint performance [12]. Likewise, the increment in the Ga, O and C content would be likely accompanied by a corresponding improvement in the impact strength of the joint. Carbon has strengthening properties, while the introduction of Gallium reduces the migration of the BM elements (mainly Al and Fe) across the joint, consequently reducing the tendency of IMC formation.

**Figure 14:**
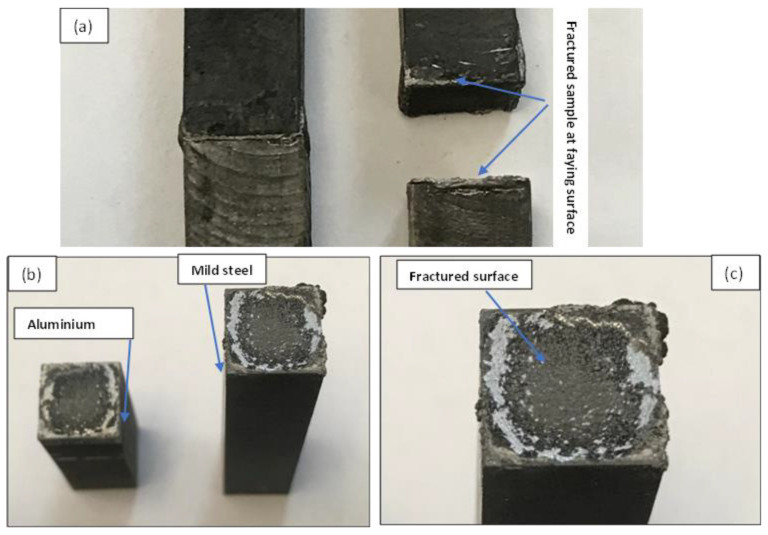
Fractured sample from the impact test: (**a**) Fractured sample at faying surface; (**b**) fractured surface of aluminium and mild steel; (**c**) close-up of the fracture surface of mild steel.

Additionally, all the bonded samples were observed to have fractured at the interface during the impact test indicating the weakest section of the joint. Figure 14 shows one of the fractured samples from the impact test.

**Figure 15:**
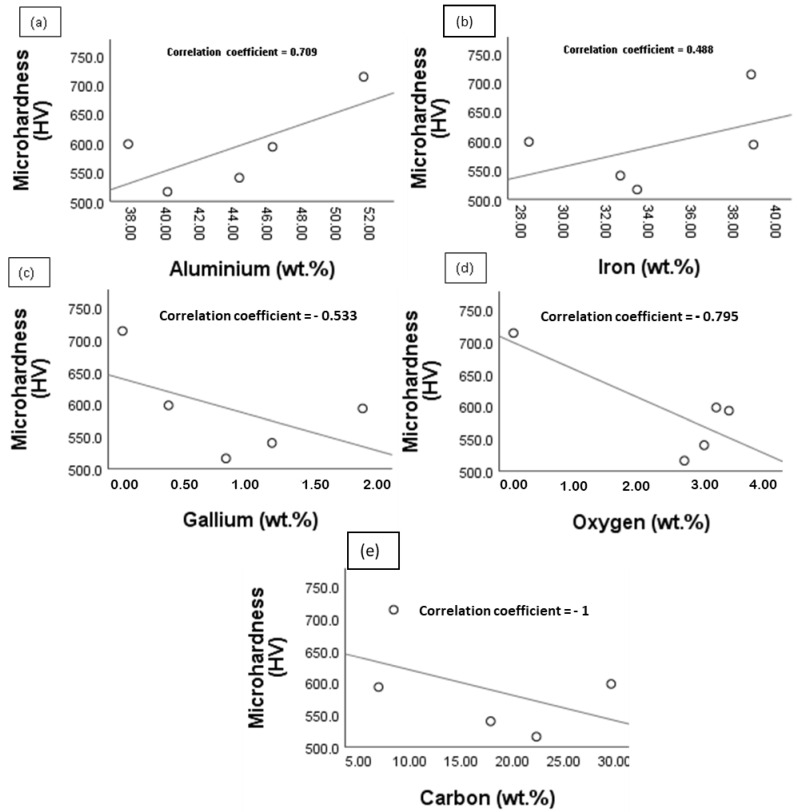
Scatter plot of the microhardness against the elemental composition (wt.%) of the reaction layer: (**a**) Microhardness against Al; (**b**) Microhardness against Fe; (**c**) Microhardness against Ga; (**d**) Microhardness against O; (**e**) Microhardness against C.

Figure 15 shows the scatter plot of the microhardness of the joint against the elemental composition of the reaction layer. Antithetical to the correlation obtained for impact strength, the Al and Fe composition (wt.%) were observed to be positively correlated with the microhardness of the reaction layer. The strengths of the correlation were found to be 0.709 and 0.488, respectively. Meanwhile, the negative correlation coefficients (−0.533, −0.795 and −1) were obtained for Ga, O and C, respectively. Correspondingly, the positive correlation coefficient obtained for Al and Fe implies a parallel increment in the microhardness of the region upon increasing the composition of these elements. This observation might also be attributed to the increase in the tendency of IMC formation. On the other hand, the increment in the Ga, O and C content would be accompanied by a reduction in the microhardness of the reaction layer.

**Figure 16:**
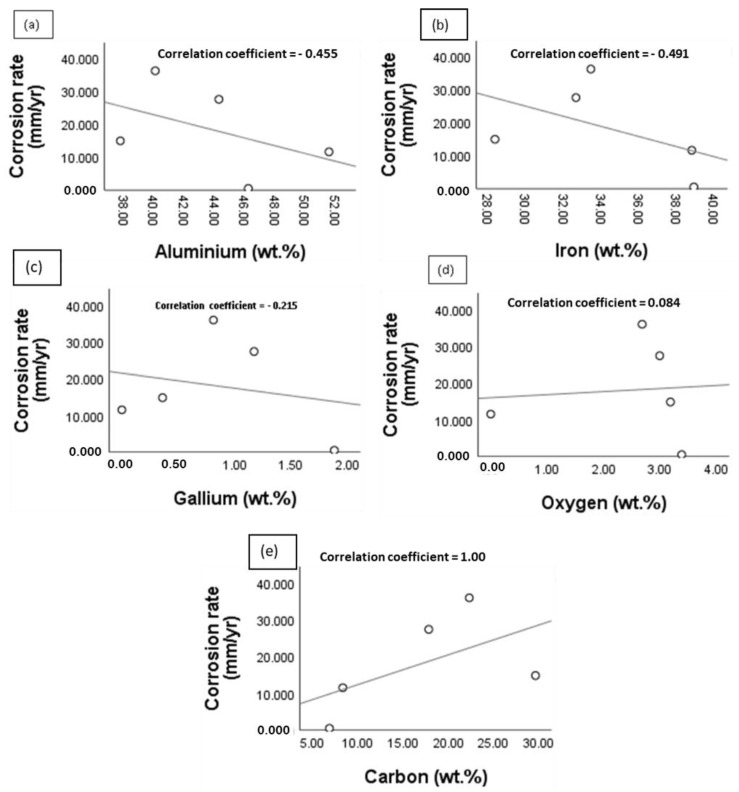
Scatter plot of the corrosion rate (mm/yr) against the elemental composition (wt.%) of the reaction layer: (**a**) corrosion rate against Al; (**b**) corrosion rate against Fe; (**c**) corrosion rate against Ga; (**d**) corrosion rate against O; (**e**) corrosion rate against C.

Figure 16 shows the scatter plot of the corrosion rate of the reaction layer against the elemental composition. The analysis revealed a negative correlation between the corrosion rate (mm/yr) and Al, Fe and Ga compositions (wt.%). The respective correlation coefficients were found to be −0.455, −0.491 and −0.215. A perfect positive correlation was also obtained for C, while a weak positive correlation (coefficient = 0.084) was obtained for O. Consequently, it can be inferred that increasing the Al, Fe and Ga content is likely to improve the corrosion property of the joint. Likewise, the corrosion behaviour would be likely impaired when the C and O contents are elevated.

### 3.3. Effect of Bonding Parameters on the Mechanical Properties of the Joint

**Figure 17:**
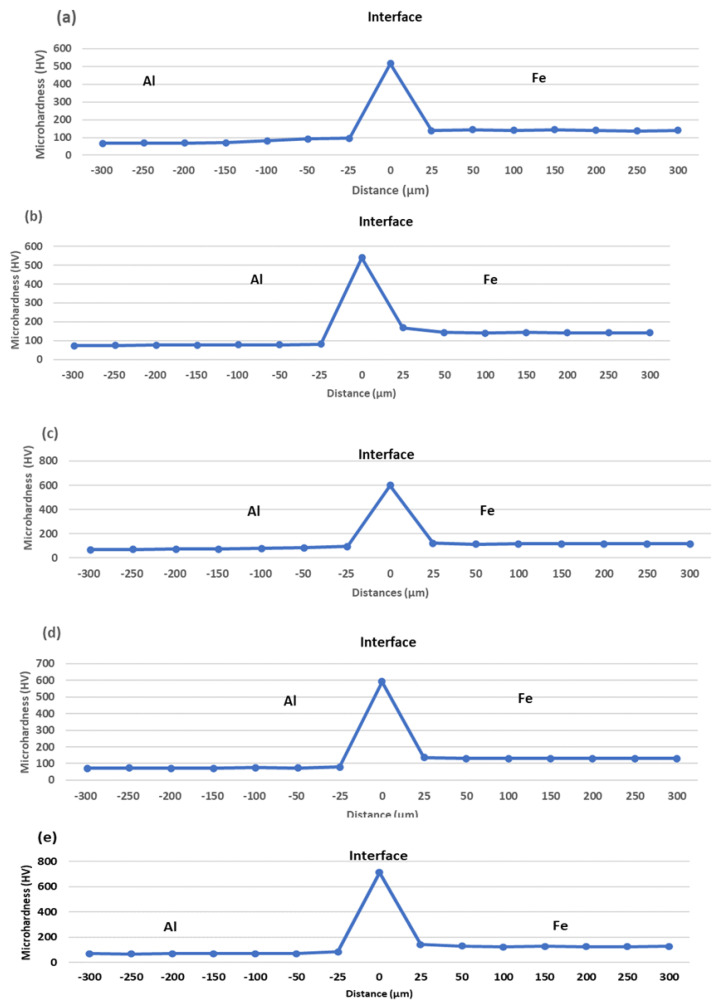
Microhardness distribution profile: (**a**) Sample E; (**b**) Sample F; (**c**) Sample G; (**d**) Sample H; (**e**) Sample I.

Expectedly, the microhardness of the aluminium BM is about 50% that of the mild steel, as shown in Table 4. However, for all samples, the highest microhardness was observed in the reaction layer, reaching a maximum of 713.5 HV in sample I. Increasing the holding time by 60 min at a constant bonding temperature (550 °C) and surface roughness (800 grit), i.e., samples E and F, resulted in a corresponding increase in the maximum microhardness of the reaction layer by 23.9 HV. Contrastingly, the impact strength decreased by 0.078 J. Figure 17a,b show the microhardness distribution profiles for samples E and F, respectively. Antithetically, a drastic reduction of 77 HV was recorded when the bonding temperature was increased by 25 °C at a constant holding time (60 min) and surface roughness (800 grit), i.e., samples E and H. This resulted in a parallel improvement in the impact strength by 0.177 J. The microhardness profile of sample H is presented in Figure 17d. An increment (81.8 HV) was also observed when the grit size was increased to 1200 (samples E and G). This led to a corresponding reduction in the impact strength by 0.186 J. Figure 17c,e present the microhardness profile of samples G and I, respectively.

**Figure 18:**
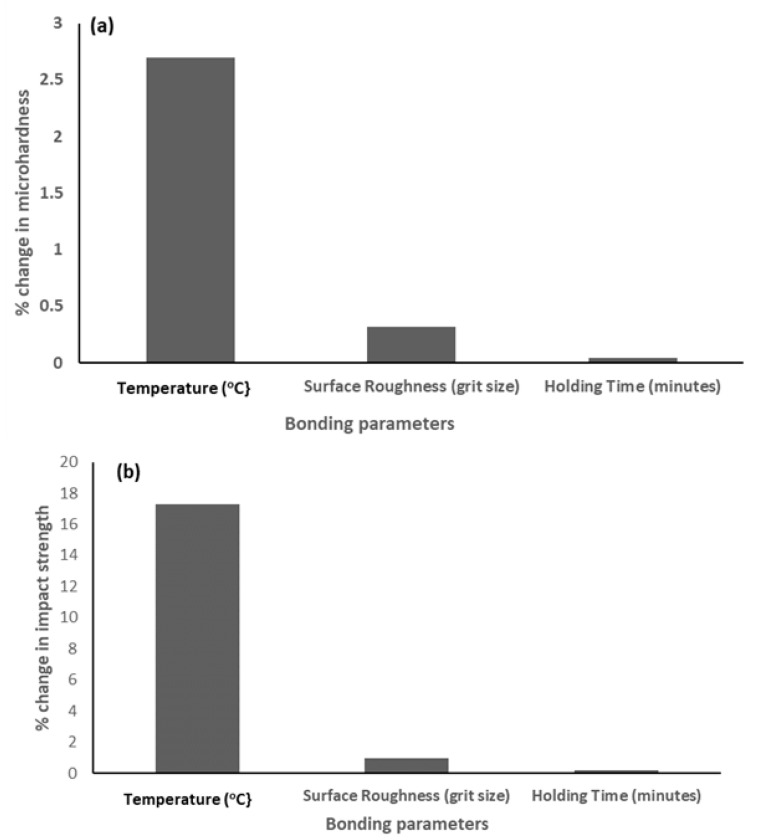
Sensitivity of the joint’s mechanical properties to 1 % change in bonding parameters: (**a**) Microhardness; (**b**) Impact strength.

The results in Table 4 revealed that sample E exhibited the optimum joint quality in terms of mechanical performance. Furthermore, it can be deduced that the maximum interfacial microhardness is negatively correlated with the bonding temperature and positively correlated with the holding time and grit size. The positive correlation might be attributed to the increase in BM composition (Al and Fe wt.%) at longer holding times and increased grit size, while the latter might be attributed to the reduction in Ga composition (wt.%) and BM composition due to increased temperature. However, a reverse trend was observed for the impact strength of the joint. Further analysis revealed that a 1% change in the bonding temperature, surface roughness (grit size) and holding time would result in 2.7%, 0.316% and 0.046% changes in the maximum interfacial microhardness. The corresponding changes in the impact strength were found to be 17.3%, 0.94% and 0.2%, respectively. This implies that the mechanical performance of the joint is most sensitive to changes in the bonding temperature and least sensitive to changes in the holding time. Figure 18 presents the sensitivity plot of the joint’s mechanical properties to changes in the bonding parameters.

### 3.4. Corrosion Behaviour of the Diffusion-Bonded BMs

**Figure 19:**
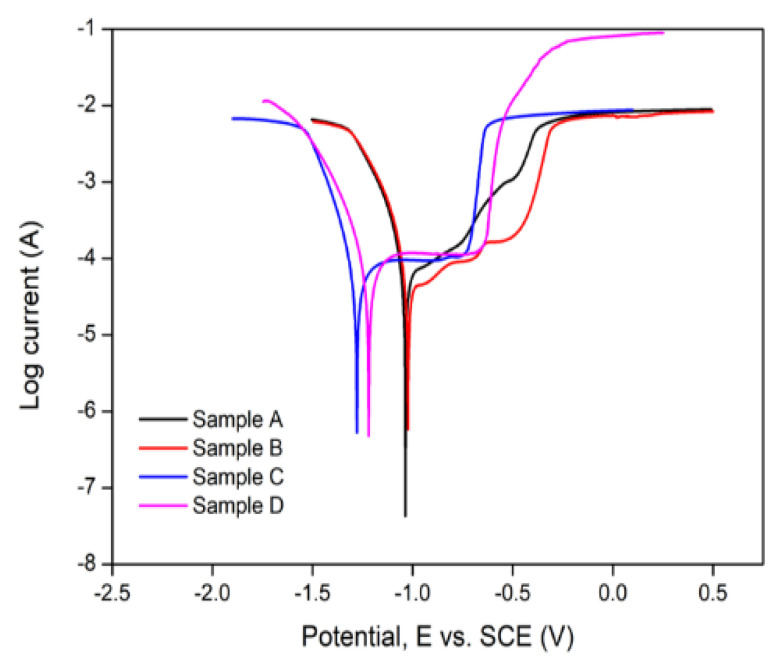
Tafel plots of samples A–D.

**Figure 20:**
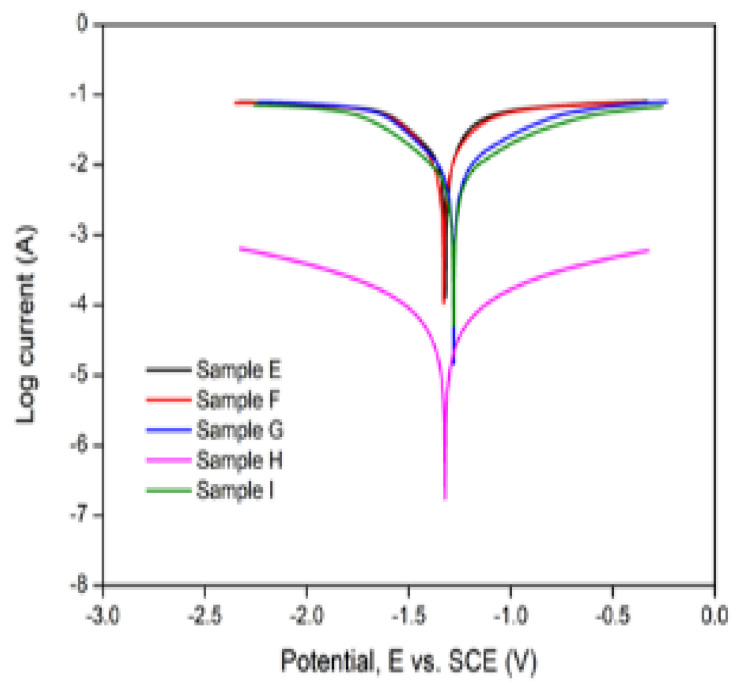
Tafel plots of samples E–I.

Table 5 presents the electrochemical parameters for the BMs and the bonded joint obtained from the potentiodynamic polarisation testing. Figure 19 presents the Tafel plot obtained for the BMs, while the Tafel plot for the bonded metals is presented in Figure 20.

Table 5 revealed that the heat-treated mild steel (sample A) has a corrosion rate of 8.1205 mm/year relative to 5.9473 mm/yr (sample B) obtained without heat treatment. Likewise, the heat-treated aluminium BM (sample C) exhibited a higher corrosion rate of 1.4302 mm/yr compared to the 0.47715 mm/yr obtained without applying heat treatment (Sample D). Consequently, the application of heat treatment resulted in a 36.5% and 199.7% increase in the rate of the degradation of mild steel and aluminium BMs, respectively, which is evident in the shift of the potential to the left in the Tafel plot (Figure 19). This implies that despite the aluminium BM exhibiting a relatively low corrosion current compared to the mild steel, the corrosion behaviour of the former is more sensitive to heat treatment than the latter. The superior corrosion properties of the aluminium BM may be attributed to their position in the galvanic series, as mild steel is relatively more susceptible to oxidation compared to the former [41]. A lower corrosion current density is attributed to the formation of corrosion products on the electrode surface, which act as barrier films to protect the surface from further degradation, thus enhancing the corrosion resistance [42]. The increase in the rate of degradation on the application of heat treatment might be attributed to the reduction in the rate of formation of these products on account of the change in BM morphology facilitated by the heat treatment process.

Meanwhile, for the bonded samples (E–I), the rate of the degradation of the joints was found to be 35.948 mm/yr, 27.38 mm/yr, 14.853 mm/yr, 0.517 mm/yr and 11.545 mm/yr for samples E–I, respectively. Sample H exhibited the least degradation rate corresponding to a current density of 0.0000445 A/cm^2^. Increasing the holding time from 60 to 120 min at a constant temperature (550 °C) and surface roughness (800 grit), i.e., samples E and F, resulted in a corresponding decrease in the degradation rate of the reaction layer. Likewise, increasing the bonding temperature from 525 to 550 °C at a constant holding time (60 min) and surface roughness (800 grit), i.e., samples E and H, resulted in a more drastic reduction in the reaction layer degradation rate. Contrastingly, comparing samples G and I, it is observed that increasing the grit size from 800 to 1200 at a constant bonding temperature (550 °C) and holding time (60 min) resulted in a corresponding increase in the interlayer degradation rate from 11.545 mm/yr to 14.853 mm/yr. Thus, it can be deduced that while the bonding temperature and holding time are negatively correlated with the corrosion rate, the grit size (surface roughness) positively correlates with the latter. The increase in the corrosion rate with an increased grit size might be attributed to an increase in the BM and Ga composition (wt.%), while its reduction owing to increased temperatures and longer holding times might be due to their reduction at the reaction layer.

**Figure 21:**
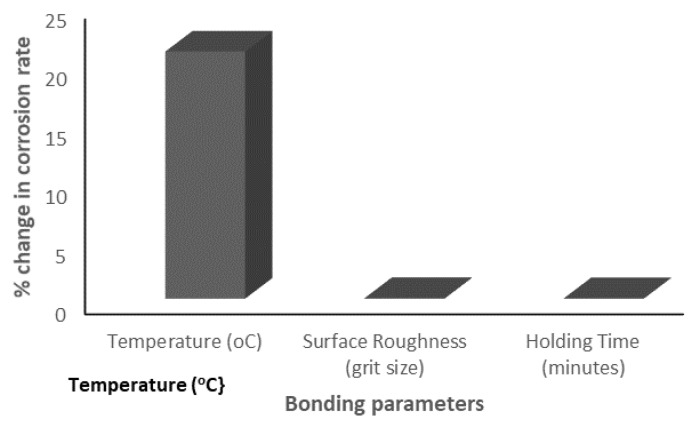
Sensitivity of the joint’s corrosion rate to 1% change in bonding parameters.

Additionally, a 1% change in the bonding temperature, surface roughness and holding time would require corresponding 20.96%, 0.057% and 0.045% changes in the corrosion rate, respectively. This implies that the interlayer degradation rate is most sensitive to the bonding temperature and least sensitive to the holding time. The sensitivity of the corrosion rate to the bonding parameters is presented in Figure 21. The effect of the gallium interlayer on the corrosion behaviour of the joints is investigated by considering samples E and I. Correspondingly, the introduction of the gallium interlayer at a constant bonding temperature (550 °C), holding time (60 min) and surface roughness (800 grit) resulted in a reduction in the degradation rate from 35.948–11.545 mm/yr. In other words, the gallium interlayer’s introduction improved the corrosion behaviour of the weld joints by 67.9%. 

## 4. Conclusions

The effect of bonding parameters on the properties of A5083 aluminium/A36 mild steel using a Ga interlayer was investigated. The bonding parameters were found to be positively correlated with the reaction layer thickness. The impact strength was negatively affected by increased interfacial microhardness, but was improved with the increased Ga, C and O (wt.%) content of the reaction layer. Likewise, excluding Ga (wt.%), the elements were also observed to be positively correlated with the corrosion rate. Furthermore, the properties of the joints were found to be most and least sensitive to the bonding temperature and holding time, respectively. Finally, the incorporation of the Ga interlayer improved the corrosion properties of the dissimilar joints by 67.9%.

## Figures and Tables

**Table 1:** Chemical composition (wt.%) of A36 mild steel and AA5083 aluminium alloy.

Material	Al	Mg	Mn	O	Cr	Si	Fe	C
AA5083	88.60	4.54	0.70	0.84	-	-	0.84	4.48
A36	-	-	0.86	-	-	0.24	93.87	5.03

**Table 2:** Sample description for the BMs and fused metal joints.

Sample Id	Material	Condition Description	Presence of Ga
A	Mild Steel	Heat treated at 550 °C for 60 min	-
B	Mild Steel	No heat treatment	-
C	Aluminium	Heat treated at 550 °C for 60 min	-
D	Aluminium	No heat treatment	
E	Fused mild steel and aluminium	Bonded at 550 °C, 60 min with surface roughness R800	Yes
F	Fused mild steel and aluminium	Bonded at 550 °C, 120 min with surface roughness R800	Yes
G	Fused mild steel and aluminium	Bonded at 550 °C, 60 min with surface roughness R1200	Yes
H	Fused mild steel and aluminium	Bonded at 525 °C, 60 min with surface roughness R800	Yes
I	Fused mild steel and aluminium	Bonded at 550 °C, 60 min with surface roughness R800	No

**Table 3:** Elemental composition of different sections of the joint.

Sample Id	Spot No.	Elemental Composition (wt%)							
		Al	Mg	O	C	Mn	Si	Ga	Fe
**E**	1	78.27	4.07	-	12.02	-	-	-	5.64
	2	40.03	0.76	2.68	22.31	-	-	0.81	33.41
	3	7.26	0.37	-	6.11	0.78	-	-	85.48
F	1	80.01	4.07	1.68	8.49	-	-	-	5.68
	2	44.25	0.72	2.99	17.83	-	0.42	1.17	32.62
	3	8.56	0.37	0.48	4.85	0.75	-	-	85.00
G	1	80.01	4.07	1.68	8.49	-	-	-	5.68
	2	44.25	0.72	2.99	17.83	-	0.42	0.36	32.62
	3	8.56	0.37	0.48	4.85	0.75	-	-	85.00
H	1	84.24	4.89	2.14	-	-	-	-	8.73
	2	46.2	1.79	3.38	6.86	1.00	-	1.88	36.88
	3	10.76	0.63	-	3.55	0.80	0.20	-	84.06
I	1	75.74	4.34	1.94	10.62	-	-	-	7.37
	2	51.55	1.34	-	8.34	-	-	-	38.77
	3	9.50	0.70	-	4.84	0.69	0.22	-	84.06

**Table 4:** Impact strength and the maximum interfacial microhardness of the bonded metal.

Sample Id	Impact Strength (J)	Maximum Interfacial Microhardness (HV)
A	-	-
B	-	152.6
C	-	-
D	-	72.7
E	0.390	516.4
F	0.312	540.3
G	0.204	598.2
H	0.213	593.4
1	0.078	713.5

**Table 5:** Electrochemical parameters that were obtained from the potentiodynamic polarisation test extracted from Tafel plots in Figure 17 and Figure 18.

Sample Id	B_a_ (V/dec)	B_c_ (V/dec)	E_corr_ (V)	I_corr_ (A)	J_corr_ (A/cm^2^)	Corrosion Rate (mm/year)	Polarisation Resistance (Ω)
A	0.193970	−0.271010	−1.033900	0.000699	0.000699	8.120500	424.070000
B	0.164900	−0.229010	−1.022400	0.000512	0.000512	5.947300	499.770000
C	0.147450	−1.304600	−1.280800	0.000123	0.000123	1.430200	586.560000
D	0.201790	0.112390	−1.318400	0.000041	0.000041	0.477150	763.450000
E	0.183520	0.138270	−1.320400	0.006187	0.003094	35.948000	5.535100
F	0.148450	0.149890	−1.331200	0.004713	0.002356	27.380000	6.873400
G	0.104380	0.151140	−1.282000	0.002557	0.001278	14.853000	10.488000
H	1.058200	1.075900	−1.319300	0.000089	0.000045	0.516550	2606.000000
I	0.120160	0.139270	−1.281900	0.001987	0.000994	11.545000	14.098000
